# Risk Factors for Rod Fracture at ≥L4-5 Levels Following Long-Segment Fusion for Adult Spinal Deformity: Results from Segment-Based Analysis

**DOI:** 10.3390/jcm14165643

**Published:** 2025-08-09

**Authors:** Se-Jun Park, Jin-Sung Park, Chong-Suh Lee, Dong-Ho Kang

**Affiliations:** 1 Department of Orthopedic Surgery, Samsung Medical Center, Seoul 06351, Republic of Korea; 2 Department of Orthopedic Surgery, Haeundae Bumin Hospital, Busan 48094, Republic of Korea

**Keywords:** adult spinal deformity, rod fracture, risk factor, at or above L4-5, segment-based analysis

## Abstract

**Background/Objectives**: Given the different biomechanical properties and surgical techniques between the L5-S1 and ≥L4-5 levels, it is necessary to explore RF risk factors at ≥L4-5 levels separately from the lumbosacral junction. This study aims to investigate the risk factors for rod fracture (RF) occurring at ≥L4-5 levels following adult spinal deformity (ASD) surgery. RF occurrence was assessed at the segment level. **Methods**: Patients who underwent ≥ 5-level fusion, including the sacrum or pelvis, with a minimum follow-up of 2 years were included in this study. Presumed risk factors in terms of patient, surgical, and radiographic variables were compared between the non-RF and RF groups at the segment level. Multivariate logistic regression analysis was performed to identify independent risk factors for RF at ≥L4-5 levels. **Results**: A total of 318 patients (mean age, 69.3 years; 88.4% female) were included, and 1082 segments were evaluated. During the mean follow-up duration of 47.4 months, RF developed in 45 (14.2%) patients for 51 (4.7%) segments. In multivariate logistic regression analysis, several risk factors were identified, as follows: the use of perioperative teriparatide (odds ratio [OR] = 0.26, *p* = 0.012), operated levels (L2-3 and L3-4 vs. L4-5 level [OR = 0.45, *p* = 0.022; OR = 0.16, *p* < 0.001, respectively]), fusion methods (posterior fusion and anterior column realignment vs. posterior lumbar interbody fusion [OR = 8.04, *p* < 0.001; OR = 5.37, *p* = 0.002, respectively]), pedicle subtraction osteotomy (PSO) (OR = 3.14, *p* = 0.020), and number of rods (four-rod configuration vs. dual-rod fixation [OR = 0.34, *p* = 0.044]). **Conclusions**: In this study, the factors related to RF at ≥L4-5 levels included the perioperative use of teriparatide, operated levels, fusion methods, performance of PSO, and rod configuration. Considering that surgical procedures vary by each segment, our findings may help establish segment-specific preventive strategies to reduce RF at ≥L4-5 levels.

## 1. Introduction

Adult spinal deformity (ASD) is a complex condition that leads to debilitating effects on both the physical and mental health of patients [1]. Previous studies have reported the effectiveness of surgical treatment for ASD and its superiority over conservative treatment [2,3]. However, since patients are typically older, and long-segment fixation is commonly required, there is a potentially increased risk of surgical complications. Pseudoarthrosis and subsequent rod fracture (RF) form one of the most common mechanical complications after surgery [4,5,6]. Despite advancements in surgical techniques and a better understanding of spinal biomechanics, RF is an ongoing issue, with rates reported up to 40% [4,5,6,7,8,9,10,11,12,13,14].

Considering the high incidence of RF, the negative impact on patient outcomes, and the associated economic burden [15], preventing RF is crucial for the success of surgical treatment for ASD. Numerous risk factors for RF have been demonstrated, including osteoporosis, obesity, increased fusion length, non-interbody fusion, residual sagittal or coronal spinal malalignment, the non-use of multiple rods, and the lumbosacral junction [7,8,9,10,11,12,13]. Due to the high vulnerability of RF at the caudal end of the construct, many studies have focused on identifying risk factors for RF at the lumbosacral junction [11,12,13,16,17,18,19,20]. However, it is reported that RF in this region accounts for only 30% of all RF cases [4,7], implying that more than two-thirds of RFs occur at segments other than the lumbosacral junction (i.e., at ≥L4-5 levels). Given the different biomechanical properties and surgical techniques between the L5-S1 and ≥L4-5 levels, it is necessary to explore RF risk factors at ≥L4-5 levels separately from the lumbosacral junction. Although previous studies have examined common risk factors for RF by lumping L5-S1 and ≥L4-5 segments together [4,5,6,7], their incorporation of the L5-S1 segment may limit their ability to specifically identify risk factors for RF at ≥L4-5 levels.

Therefore, this study aims to investigate the risk factors for developing RF at ≥L4-5 levels following long-segment fusion for ASD. Considering that fusion techniques may vary by segment, even in the same patient, RF occurrence was assessed at the segment level as well as at the patient level.

## 2. Materials and Methods

### 2.1. Study Design and Cohort

This study was approved by our institutional review board (IRB no. 2024-07-144; approved on 1 August 2024). The acquisition of informed consent was waived due to the retrospective nature of this study. We retrospectively analyzed patient records from a prospective ASD database at our academic institution. Consecutive patients who underwent long-level fusion for degenerative ASD between 2013 and 2022 were enrolled in this study. The inclusion criteria were as follows: (1) ASD, defined as pelvic incidence minus lumbar lordosis (PI-LL) mismatch ≥ 10°, pelvic tilt (PT) ≥ 25°, thoracic kyphosis (TK) ≥ 60°, C7–sagittal vertical axis (C7–SVA) ≥ 5 cm, or a coronal Cobb angle ≥ 30°; (2) ≥5-level fusion including the sacrum or pelvis; and (3) a minimum follow-up of 2 years. Patients were excluded if they had previous pan-lumbar arthrodesis; if they had neuromuscular, inflammatory, or other non-degenerative pathological conditions; or if they had incomplete radiographic data. Patients with RFs at L5-S1 were also excluded, as these may alter the likelihood of RF occurring at ≥L4-5 levels.

The primary indication for surgery was sagittal imbalance presenting as postural stooping and back pain. The surgical strategy was tailored to each segment; for L5-S1 lesions, ALIF or PLIF was performed, while for lumbar levels above L5, ACR was selectively applied to segments according to the preoperative corrective plan. Iliac screw fixation was routinely used, except in cases with a previously fused L5-S1 segment.

### 2.2. Selection of Segments for Evaluation

**Figure 1:**
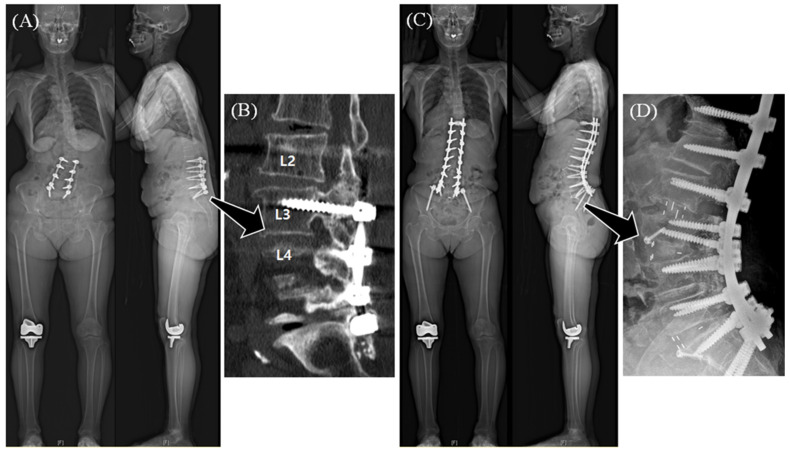
(**A**) A case of a 79-year-old female with iatrogenic flatback deformity. (**B**) A solid posterior fusion mass across L2 to L5 was seen in the preoperative computed tomography scan. (**C**) Revisional deformity correction was performed through a posterior–anterior–posterior approach. (**D**) Fusion mass osteotomy was performed, followed by lateral lumbar interbody fusion and anterior column realignment for the L2-3 and L3-4 levels, respectively. No additional fusion procedure was performed for the L4-5 level. Therefore, only the L2-3 and L3-4 levels were included in the analysis, while the L4-5 segment was not.

**Figure 2:**
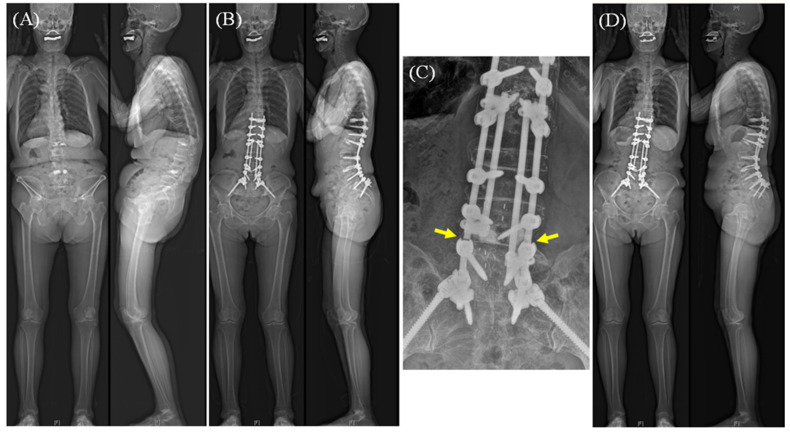
(**A**) A case of a 72-year-old female with severe thoracolumbar kyphosis. (**B**) Corrective surgery was performed using anterior column realignment at the L3-4 and L4-5 levels, and pedicle subtraction osteotomy at the L2 vertebra. Two satellite rods were inserted, covering from T12-L1 to L4-5 on the left side and from T12-L1 to L5-S1 on the right side. Therefore, the L1-2, L2-3, and L3-4 levels were considered to be reinforced by 4 rods, while the L4-5 segment was reinforced by 3 rods. (**C**) Two years after surgery, bilateral rod fractures developed at the L4-5 segment (arrows). (**D**) Despite bilateral rod fractures, revision surgery was not performed because global alignment had remained relatively stable without symptom worsening.

The primary outcome of this study was the occurrence of RF evaluated at the segment level. All segments that received fusion surgery for naïve segments were included in the evaluation. Therefore, segments identified as already fused (confirmed on preoperative computed tomography [CT]) due to prior fusion procedures were excluded from the analysis. However, segments with prior fusion surgery were included in the analysis if they underwent the following types of revision procedures: (1) segments with prior posterior fusion where the posterior fusion mass was broken down and correction was re-performed through the anterior approach (Figure 1) and (2) segments with non-union after prior interbody fusion where cage removal and revisional interbody fusion were performed, either anteriorly or posteriorly.

### 2.3. Outcome Measure

RF was diagnosed when the breakage of rods was found in follow-up plain radiographs or CT scans. Based on RF occurrence, all segments were divided into non-RF and RF groups. In addition to segment-based analysis of RF, unilaterality, bilaterality, and performance of revision surgery were evaluated at the patient level. The decision to perform revision surgery was multifactorial, based on a combination of clinical, radiological, and patient-centered factors, and was generally considered after conservative management failed. Key indications for revision included persistent or recurrent pain, the development of a new neurological deficit, progressive alignment deterioration, fracture characteristics indicating instability (particularly bilateral or displaced fractures), and definitive evidence of pseudarthrosis at the fracture level. Furthermore, a marked decline in a patient’s ability to perform daily activities attributed to the fracture was also a key consideration. Risk factor analyses were performed at the segment level.

### 2.4. Presumed Risk Factors

Presumed risk factors were compared between the groups in terms of patient, surgical, and radiographic factors. Patient factors included sex, age, American Society of Anesthesiologists (ASA) physical status, body mass index (BMI), T-score (lowest values in spine or hip bone densitometry), perioperative administration of teriparatide (only for cases with ≥ 3-month use perioperatively), and follow-up duration. In this study cohort, teriparatide was selectively administered to patients with osteoporosis (T-score < −2.5 in spine or hip bone densitometry) before 2019. Since then, the indication of teriparatide was expanded to patients with osteopenia (T-score < −1.0) or those with a history of osteoporotic compression fractures. Only patients who received teriparatide for a total duration of at least three months, including both preoperative and postoperative periods, were included. Surgical factors included operated levels (L4-5, L3-4, L2-3, and L1-2), fusion methods (posterior lumbar interbody fusion [PLIF], lateral lumbar interbody fusion [LLIF], anterior column realignment [ACR], and posterior fusion [PF]), PSO, corpectomy, number of rods (2, 3, or 4), pelvic fixation, and fusion length. The rod materials were all titanium. For the assessment of rod configuration, only the number of rods covering the segment was counted (Figure 2). For radiographic factors, postoperative sagittal and coronal parameters were measured 6 weeks postoperatively as follows: PI-LL, sacral slope (SS), PT, T1 pelvic angle (T1PA), C7–SVA, and C7–center sacral vertical line (C7–CSVL). In addition to these conventional radiographic parameters, the correction statuses were evaluated using the global alignment assessment metrics: Scoliosis Research Society (SRS)-Schwab sagittal modifiers for PI-LL, PT, and SVA (classified as grade 0, +, and ++), Global Alignment and Proportion (GAP) score (classified as proportioned, moderately proportioned, and severely proportioned), and restoration relative to Roussouly curve types [21,22,23]. The Roussouly type was determined using methods previously published by Pizones [23]. The first group was categorized as “theoretical” Roussouly types according to PI values: type 1: PI < 45°, LL apex at or below the L4-5 space; type 2: PI < 45°, LL apex above the L4; type 3: 45° ≤ PI < 60°; and type 4: PI ≥ 60°. The second group was categorized as “current” Roussouly types: type 1: SS < 35°, LL apex at or below the L4-5 space; type 2: SS < 35°, LL apex above the L4; type 3: 35° ≤ SS < 45°; and type 4: SS ≥ 45°. If the theoretical and current Roussouly types matched, the sagittal alignment was considered appropriately restored according to the Roussouly curve type.

### 2.5. Statistical Analysis

All data were presented as numbers with percentages for categorical variables and as means with standard deviations for continuous variables. A paired *t*-test was performed to compare preoperative and postoperative 6-week sagittal parameters. Risk factor analyses involved two steps: (1) comparing presumed risk factors between the non-RF and RF groups using independent *t*-tests (or Mann–Whitney U tests, as appropriate) for continuous variables and chi-square tests for categorical variables, and (2) performing multivariate logistic regression. To build the multivariate model, variables with a *p*-value < 0.10 in the bivariate analysis were selected as covariates. We confirmed that there was no significant multicollinearity among these selected variables (e.g., variance inflation factor < 10). These covariates were entered into the final logistic regression model to identify independent risk factors. All statistical analyses were performed using IBM SPSS Statistics for Windows (version 27; IBM Corp., Armonk, NY, USA). Statistical significance was set at *p* < 0.05.

## 3. Results

### 3.1. Baseline Data

A total of 318 patients were included in this study (female, 88.4%; age, 69.3 years; BMI, 25.9 kg/m^2^; T-score, −1.4) (Table 1). The ASA physical status showed that 5.7%, 79.2%, and 15.1% of the patients had grades 1, 2, and 3, respectively. Teriparatide was administered perioperatively to 52 patients (16.4%). The mean duration of teriparatide use was 3.8 ± 1.2 months and 5.0 ± 1.8 months before and after surgery, respectively. Previous fusion surgery had been performed in 128 patients (40.3%). The surgical techniques applied varied significantly across segments within the same patient. Of the 318 patients, only 23 (7.2%) underwent a single type of fusion procedure for all evaluated levels. The remaining 295 patients (92.8%) were treated with a combination of at least two different fusion methods. The main corrective technique was the anterior–posterior combined approach, which was performed on 74.5% of the patients. PSO and corpectomy were conducted in 10.1% and 6.3% of the patients, respectively. The mean number of interbody fusion levels was 3.6. The mean fusion length was 7.3. Satellite rods were used in 100 patients (31.5%), and pelvic fixation was performed in 73.6% of the patients. The mean follow-up duration was 47.4 months. All radiographic parameters, including PI-LL, SS, PT, T1PA, C7–SVA, and C7–CSVL, significantly improved after surgery: PI-LL from 41.3° to 6.6°; SS from 20.9° to 35.3°; PT from 32.9° to 18.8°; T1PA from 32.9° to 15.6°; C7–SVA from 80.3 mm to 20.4 mm; and C7–CSVL from 17.4 mm to 12.7 mm.

### 3.2. Incidence RF and Subsequent Revision Surgery

In total, 45 (14.2%) of the 318 patients had RF at ≥L4-5 levels (Table 2). Unilateral RF developed in 13 patients and bilateral RF developed in 32 patients (same level in 24 patients and different levels in 14 patients). Among the 32 patients with bilateral RF, 21 underwent revision surgery. In the segment-based analysis, a total of 1082 segments were included, with 223 segments at L4-5, 264 at L3-4, 291 at L2-3, and 304 at L1-2. Among all the segments evaluated, RF was found in 51 segments, with the highest rate in the L4-5 (8.1%, 18/223), followed by L3-4 (4.9%, 13/264), L2-3 (4.8%, 14/291), and ≥L1-2 (2.0%, 6/304) (*p* = 0.013, not shown in the table). Revision surgery was performed more frequently at L4-5, followed by L3-4, L2-3, and L1-2, without a significant difference (*p* = 0.331).

### 3.3. Comparison of Variables Between the Groups

With regard to patient factors, sex, age, ASA grades, and BMI were not significantly different between the two groups (Table 3). The T-score was lower in the RF group than in the non-RF group, albeit without significance (−1.84 vs. −1.47, *p* = 0.080). Teriparatide was administered significantly more to the patients in the non-RF group than to those in the RF group (18.4% vs. 7.8%, *p* = 0.045). RF rates significantly differed across the operated levels: the RF group included significantly more L4-5 levels and significantly fewer L1-2 levels than the non-RF group (35.3% vs. 19.9% for L4-5, 11.8% vs. 28.9% for L1-2, *p* = 0.013). Regarding fusion methods, ACR and PF were performed more frequently in the RF group than in the non-RF group (37.3% vs. 19.1% for ACR, 41.2% vs. 29.9% for PF), while PLIF and LLIF were performed more frequently in the non-RF group than in the RF group (17.0% vs. 9.8% for PLIF, 34.0% vs. 11.8% for LLIF), with their differences significant (*p* < 0.001). The RF group included more cases with PSO than the non-RF group (13.7% vs. 5.2%, *p* = 0.010). Regarding the number of rods, significantly more patients in the non-RF group underwent satellite-rod fixation than those in the RF group (30.6% vs. 14.7%, *p* = 0.043). Pelvic fixation did not differ between the groups. Fusion length was significantly greater in the RF group than in the non-RF group (8.0 vs. 7.3 levels, *p* = 0.006). All radiographic factors showed no significant differences between the non-RF and RF groups regarding postoperative radiographic parameters, including PI-LL, SS, PT, T1PA, C7–SVA, and C7–CSVL, the SRS-Schwab sagittal modifiers for PI-LL, PT, and SVA, GAP score categories, and restoration relative to Roussouly curve type (Table 4).

### 3.4. Multivariate Logistic Regression Analysis

Variables with *p*-values < 0.10 in the bivariate comparisons, including T-score, perioperative use of teriparatide, operated levels, fusion methods, performance of PSO, number of rods, and total fusion length, were submitted to multivariate logistic regression analysis. In the multivariate analysis, we identified five independent risk factors for RF at ≥L4-5: perioperative use of teriparatide, operated levels, fusion methods, PSO, and number of rods (Table 5). Perioperative administration of teriparatide significantly reduced the risk of RF (odds ratio [OR] = 0.30, *p* = 0.031). The L1-2 and L2-3 segments were associated with a significantly lower risk of RF compared to L4-5 (OR = 0.16, *p* = 0.006 for ≥L1-2; OR = 0.45, *p* = 0.022 for L2-3). ACR and PF significantly increased the risk of RF compared with PLIF (OR = 5.37, *p* = 0.002 for ACR; OR = 8.04, *p* < 0.001 for PF). PSO significantly increased the risk of RF (OR = 3.14, *p* = 0.020). Regarding the number of rods, the four-rod configuration significantly decreased the risk of RF compared to dual-rod fixation (OR = 0.34, *p* = 0.044).

## 4. Discussion

Given the extensive nature of surgical procedures, including longer fusion lengths and various degrees of osteotomies, ASD surgery is bound to carry significant risks of mechanical complications, such as RF. The current literature documents several related factors in terms of patient, surgical, and radiographic aspects [7,8,9,10,11,12,13]. However, mixing the L5-S1 and ≥L4-5 levels in analyses may limit the ability to predict RF confined to the ≥L4-5 levels, as the lumbosacral junction has distinct biomechanical properties, including caudal inclination of the disc space and the greatest mechanical load compared with other segments of the spine. Therefore, this study exclusively focused on ≥L4-5 levels. Most previous studies have been conducted at the individual “patient” level. Such patient-level analysis can be easily applicable to the lumbosacral junction because every patient has only one L5-S1 segment. However, this patient-level approach may be limited for segments at ≥L4-5 because fusion methods usually vary by segment, even in the same patient. Additionally, even if multi-rod constructs are used, they may not uniformly cover all instrumented segments, resulting in some segments not being covered by satellite rods (Figure 2). Accordingly, we believe that segment-level analysis is helpful to identify the distinct risk factors for RF at ≥L4-5 levels.

**Figure 3:**
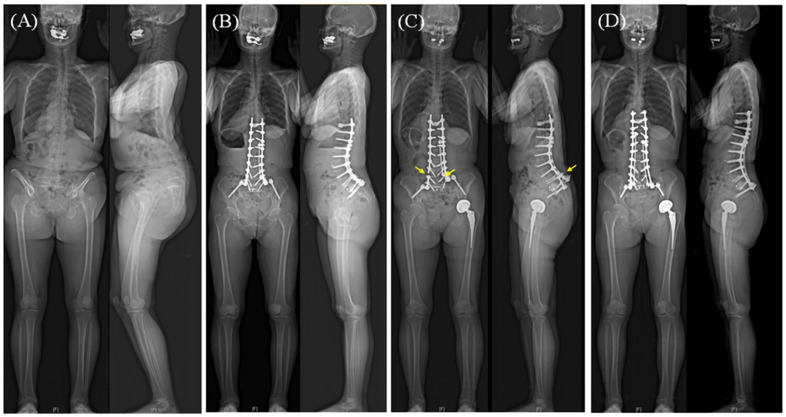
(**A**) A case of a 69-year-old female with severe flatback deformity. (**B**) Corrective surgery was performed through anterior column realignment at the L2-3 and L3-4 levels, with a satellite rod covering from the L1-2 to the L4-5 levels on the right side. The L4-5 level was treated by posterior fusion without anterior support. (**C**) One year after surgery, instrumentation failure developed, including left-side rod fracture at the L4-5 level, breakage of the right L5 pedicle screw, and fixation loss distal to the L4-5 level (arrows). (**D**) Revision surgery was performed by adding lateral lumbar interbody fusion at the L4-5 level and fixing the failed instrumentation.

In this study, five independent risk factors were identified: perioperative use of teriparatide, operated levels, fusion methods, performance of PSO, and dual-rod fixation (Table 5). Among these, PSO and dual-rod fixation are well-documented risk factors for RF following ASD surgery [7,8,10,24,25,26,27]. However, we found that only the four-rod configuration was effective in preventing RF, whereas the three-rod configuration did not significantly decrease the risk of RF (Figure 2). The lack of statistical significance for the three-rod construct should be interpreted cautiously. Descriptively, the fracture rate in this group was low, with only 4 of 135 segments (3.0%) experiencing a fracture. However, this small absolute number of events limited the statistical power of the analysis. Consequently, a potential protective effect from a three-rod construct cannot be excluded based on these results. We observed that the risk of RF varied by operated segments, being higher at the L4-5 and L3-4 levels compared to the L1-2 or L2-3 levels. This finding may be explained by the fact that, just as L5-S1 is identified as a common site for RF, the L4-5 and L3-4 levels are subjected to higher mechanical stress than the L2-3 and L1-2 levels (Figure 2). The finding that fusion length was significantly longer in the RF group than in the non-RF group in the bivariate comparison may support this interpretation (Table 4). Another possible reason may be related to the rod bending for sagittal correction. As the L3-4 and L4-5 levels commonly correspond to the apex of the sagittal contour, a high degree of mechanical stress may be concentrated in these segments. Additionally, the repeated bending procedures may weaken the strength of the rods, causing stress to increase. Concerning fusion methods, segments treated by PF (i.e., no anterior support) or ACR were associated with an increased risk of RF compared to the PLIF and LLIF procedures. It is generally accepted that posterior fusion without interbody support raises the risk of pseudoarthrosis and RF, as shown in Figure 3 [28]. ACR, a recently introduced procedure, allows for powerful restoration of the segmental angle in lumbar kyphotic deformities [29]. The ACR technique includes the release of the anterior longitudinal ligament followed by the insertion of hyperlordotic cages. The elimination of the anterior tension band of segment function and additional posterior column osteotomies may render the ACR segments unstable, potentially leading to pseudoarthrosis and RF (Figure 2). Consistent with our findings, previous studies have shown a relatively high incidence of RF following the ACR procedure in ASD surgery [9,30]. Lastly, we found that perioperative use of teriparatide significantly decreased the risk of RF, with an OR of 0.30. This may be attributed to teriparatide’s anabolic pharmacological mechanism of action as a fusion agent. This is supported by the fact that teriparatide is associated with faster dynamic bone formation, which was significant after three months of administration and reached a peak at four months, with a 6-fold increase compared to no treatment in histomorphometry analysis [31]. Agreeing with our findings, a recent study by Mohanty et al. reported a positive impact of teriparatide on fusion, showing that teriparatide treatment for patients with osteopenia lowered rates of symptomatic pseudoarthrosis following ASD surgery [32].

It is noteworthy that postoperative alignment status did not significantly influence RF occurrence at ≥L4-5 levels. This finding can be explained by several points. First, the sagittal alignment was adequately restored postoperatively in the majority of the patients, resulting in a relatively low proportion of patients with postoperative severe malalignment even within the RF group. Second, the impact of postoperative sagittal alignment on developing RF might differ between the L5-S1 and ≥L4-5 levels. Theoretically, proper restoration of sagittal alignment is beneficial in preventing mechanical complications because inappropriate alignment may disrupt stable load transitions across the operated segments, potentially provoking RF. This concept has been well proven in lumbosacral junction analysis, showing that proper correction of sagittal alignment is associated with a decreased pseudoarthrosis rate at the L5-S1 level [11,18,33,34,35]. However, the literature inconsistently reports the role of sagittal alignment in developing RF when including both the L5-S1 and ≥L4-5 levels together. Sardi et al. reported that greater postoperative PT was associated with an increased risk of RF [4]. However, since their study included all instrumented segments and lumped the L5-S1 and ≥L4-5 levels, it remains unclear if this result can be equally applied exclusively for ≥L4-5 levels. Other studies have reported that more severe baseline sagittal malalignment and greater sagittal alignment correction are associated with RF occurrence [24,36]; however, these studies failed to demonstrate that postoperative sagittal alignment influenced the development of RF. In accordance with our findings, Marques et al. showed that RF was not associated with postoperative alignment status but with varying surgical techniques, such as increased fusion length, posterior-only surgical approaches, and PSO [7]. Our findings imply that local factors (e.g., operated level, fusion methods, PSO, and number of rods) may play a more significant role than sagittal alignment status. Although sagittal alignment was not identified as a risk factor in our study, achieving proper sagittal alignment remains essential for minimizing L5-S1 pseudoarthrosis, preventing proximal junctional complications, and improving clinical symptoms.

Summarizing our results, we recommend the following strategies to mitigate the risk of RF at ≥L4-5 levels: (1) perform interbody fusion at L4-5 whenever feasible; (2) consider teriparatide routinely for all patients with osteopenia, or at least for patients undergoing high-risk procedures, such as ACR or PSO; and (3) use satellite rods with the four-rod configuration covering at-risk segments, such as L4-5, ACR, and PSO.

This study has some limitations. First, the retrospective design is an inherent limitation despite using a prospectively collected dataset. Second, since we analyzed radiographically diagnosed RFs, a significant number of asymptomatic unilateral RFs were included. However, since these fractures can be recognized as precursors to symptomatic cases eventually requiring revision surgery, we believe that all RFs are clinically important. A future study with longer follow-up is needed to determine the outcome of these unilateral RFs. Third, the study cohort included patients whose UIV was primarily located in the lower thoracic or upper lumbar spine (mean fusion length, 7.3). Therefore, our results may have limited applicability to patients who have undergone fusion involving the upper thoracic spine. Fourth, since not all cases of radiographic nonunion result in RF, the diagnosis of RF does not fully capture all pseudoarthrosis cases, potentially underestimating the actual incidence of pseudoarthrosis. However, the radiographic assessment of solid fusion (or pseudoarthrosis) in operated segments is also incomplete for accurately predicting RF, as RF can develop even in cases of apparent solid fusion on radiographs [37]. Fifth, while our study analyzed overall postoperative sagittal alignment, it did not include a specific subgroup analysis comparing overcorrected versus undercorrected patients. However, given that no significant differences were found in any of the comprehensive alignment metrics evaluated, including GAP scores and SRS-Schwab modifiers, we believe this omission does not alter our main conclusions. Sixth, we acknowledge that surgeon experience and surgical techniques, such as proper endplate preparation and instrument handling, may have a significant impact on developing pseudoarthrosis [38]. Seventh, this study did not assess preoperative paraspinal muscle quality, such as fatty infiltration or muscle mass, which is a potential confounding variable that could influence spinal stability and the risk of mechanical failure. Furthermore, the absence of a systematic CT-based assessment of pseudarthrosis is a significant limitation. Finally, our statistical analysis treated each segment as an independent observation. This approach does not account for the potential correlation of multiple segments within the same patient, which may lead to an underestimation of standard errors and potentially inflated statistical significance. However, as this limitation primarily affects the precision of the estimates (i.e., *p*-values and confidence intervals) rather than the direction and magnitude of the effect sizes (i.e., OR), the overall clinical conclusions of our study regarding the key risk factors remain robust. Nevertheless, the results should be interpreted with this limitation in mind.

## 5. Conclusions

In this study, RF developed in 45 (14.2%) of 318 patients and in 51 (4.7%) of 1082 segments at ≥L4-5 levels. Related factors included perioperative use of teriparatide, operated levels, fusion methods, performance of PSO, and rod configuration. Considering the surgical procedures vary by each segment, our findings may help establish level-specific strategies to reduce RF.

## Figures and Tables

**Table 1:** Baseline data of the total cohort (N = 318).

Variables	Statistics
Sex (female), n (%)	281 (88.4%)
Age (years), mean (SD)	69.3 (6.7)
ASA physical status	
Grade 1, n (%)	18 (5.7%)
Grade 2, n (%)	252 (79.2%)
Grade 3, n (%)	48 (15.1%)
BMI (kg/m^2^), mean (SD)	25.9 (4.0)
T-score, mean (SD)	−1.4 (1.4)
Use of teriparatide, n (%)	52 (16.4%)
Previous fusion, n (%)	128 (40.3%)
Anterior–posterior combined approach, n (%)	237 (74.5%)
PSO, n (%)	32 (10.1%)
Corpectomy, n (%)	20 (6.3%)
Interbody fusion levels, mean (SD)	3.6 (1.0)
Fusion length, mean (SD)	7.3 (1.9)
Number of rods	
2, n (%)	218 (68.5%)
3, n (%)	39 (12.3%)
4, n (%)	61 (19.2%)
LIV	
S1, n (%)	84 (26.4%)
Pelvis, n (%)	234 (73.6%)
Follow-up (months), mean (SD)	47.4 (22.1)

ASA, American Society of Anesthesiologists; BMI, body mass index; PSO, pedicle subtraction osteotomy; LIV, lowest instrumented vertebra.

**Table 2:** Incidence of RF and revision surgery.

	RF	Revision Surgery
Patient level (N = 318), n (%)	45 (14.2%)	21 (6.6%)
Segment level (N = 1082), n (%)	51 (4.7%)	25 (2.3%)
L4-5 (N = 223), n (%)	18 (8.1%)	7 (3.1%)
L3-4 (N = 264), n (%)	13 (4.9%)	7 (2.7%)
L2-3 (N = 291), n (%)	14 (4.8%)	8 (2.7%)
L1-2 (N = 304), n (%)	6 (2.0%)	3 (1.0%)

RF, rod fracture.

**Table 3:** Comparison of patient and surgical factors between the two groups.

Variables	Non-RF Group (N = 1031)	RF Group (N = 51)	*p*
***Patient factors***			
Sex (female), n (%)	920 (89.2%)	42 (82.4%)	0.127
Age (years), mean (SD)	69.4 (6.4)	68.4 (7.7)	0.285
ASA physical status			0.280
Grade 1, n (%)	62 (6.0%)	3 (5.9%)	
Grade 2, n (%)	829 (80.4%)	45 (88.2%)	
Grade 3, n (%)	140 (13.6%)	3 (5.9%)	
BMI (kg/m^2^), mean (SD)	25.8 (3.8)	25.2 (3.6)	0.265
T-score on bone densitometry, mean (SD)	−1.47 (1.44)	−1.84 (1.39)	0.080
Use of teriparatide, n (%)	190 (18.4%)	4 (7.8%)	**0.045**
***Surgical factors***			
Operated segments			**0.013**
L4-5, n (%)	205 (19.9%)	18 (35.3%)	
L3-4, n (%)	251 (24.3%)	13 (25.5%)	
L2-3, n (%)	277 (26.9%)	14 (27.5%)	
L1-2, n (%)	298 (28.9%)	6 (11.8%)	
Fusion methods			**<0.001**
PLIF, n (%)	175 (17.0%)	5 (9.8%)	
LLIF, n (%)	351 (34.0%)	6 (11.8%)	
ACR, n (%)	197 (19.1%)	19 (37.3%)	
PF, n (%)	308 (29.9%)	21 (41.2%)	
PSO, n (%)	54 (5.2%)	7 (13.7%)	**0.010**
Corpectomy, n (%)	32 (3.1%)	3 (5.9%)	0.274
Number of rods spanning each segment			**0.043**
Two, n (%)	715 (69.4%)	44 (86.3%)	
Three, n (%)	131 (12.7%)	4 (7.8%)	
Four, n (%)	185 (17.9%)	3 (5.9%)	
Pelvic fixation, n (%)	790 (76.6%)	40 (78.4%)	0.766
Fusion length, mean (SD)	7.3 (1.9)	8.0 (1.9)	**0.006**
Follow-up duration, mean (SD)	47.7 (22.3)	50.7 (22.3)	0.349

Bold *p*-values indicate statistical significance. ASA, American Society of Anesthesiologists; BMI, body mass index; PLIF, posterior lumbar interbody fusion; LLIF, lateral lumbar interbody fusion; ACR, anterior column realignment; PF, posterior fusion; PSO, pedicle subtraction osteotomy.

**Table 4:** Comparison of radiographic risk factors between the two groups.

Variables	Non-RF Group (N = 1031)	RF Group (N = 51)	*p*
***Postoperative radiographic parameters***			
PI-LL (°), mean (SD)	6.1 (11.2)	6.6 (14.9)	0.760
SS (°), mean (SD)	35.1 (9.3)	35.9 (8.9)	0.550
PT (°), mean (SD)	18.4 (8.9)	18.6 (11.8)	0.873
T1PA (°), mean (SD)	15.2 (8.6)	15.0 (10.3)	0.882
C7–SVA (mm), mean (SD)	19.0 (31.5)	13.3 (32.4)	0.203
C7–CSVL (mm), mean (SD)	12.6 (10.8)	13.9 (11.5)	0.400
***SRS-Schwab PI-LL sagittal modifier***			0.589
Grade 0 (<10°), n (%)	698 (67.7%)	32 (62.7%)	
Grade + (10–20°), n (%)	218 (21.1%)	11 (21.6%)	
Grade ++ (>20°), n (%)	115 (11.2%)	8 (15.7%)	
***SRS-Schwab PT sagittal modifier***			0.358
Grade 0 (<20°), n (%)	586 (56.8%)	24 (47.1%)	
Grade + (20–30°), n (%)	359 (34.8%)	21 (41.2%)	
Grade ++ (>30°), n (%)	86 (8.3%)	6 (11.8%)	
***SRS-Schwab SVA sagittal modifier***			0.624
Grade 0 (<4 cm), n (%)	767 (74.4%)	40 (78.4%)	
Grade + (4.0–9.5 cm), n (%)	250 (24.2%)	11 (21.6%)	
Grade ++ (>9.5 cm), n (%)	14 (1.4%)	0 (0.0%)	
***GAP score***			0.197
Proportioned, n (%)	296 (28.7%)	9 (17.6%)	
Moderately disproportioned, n (%)	484 (46.9%)	26 (51.0%)	
Severely disproportioned, n (%)	251 (24.3%)	16 (31.4%)	
***Roussouly curve type***			0.371
Restored, n (%)	602 (58.4%)	33 (64.7%)	
Non-restored, n (%)	429 (41.6%)	18 (35.3%)	

PI, pelvic incidence; LL, lumbar lordosis; SS, sacral slope; PT, pelvic tilt; T1PA, T1 pelvic angle; C7–SVA, C7–sagittal vertical axis; C7–CSVL, C7–center sacral vertical line; SRS, Scoliosis Research Society; GAP, Global Alignment Proportion.

**Table 5:** Multivariate logistic regression analysis for independent risk factors for RF at ≥L4-5 levels.

Variables	Odds Ratio	95% Confidence Interval	*p*
T-score on bone densitometry	0.90	0.73–1.11	0.326
Perioperative use of teriparatide	0.30	0.10–0.89	**0.031**
Operated levels			
L4-5	Reference		
L3-4	0.57	0.26–1.25	0.161
L2-3	0.45	0.20–0.87	**0.022**
L1-2	0.16	0.04–0.59	**0.006**
Fusion methods			
PLIF	Reference	-	-
LLIF	1.07	0.31–3.75	0.914
ACR	5.37	1.83–15.79	**0.002**
PF	8.04	2.28–25.04	**<0.001**
PSO	3.14	1.19–8.23	**0.020**
Number of rods			
Two	Reference		
Three	0.35	0.11–1.15	0.085
Four	0.34	0.12–0.97	**0.044**
Total fusion lengths	1.11	0.96–1.28	0.156

Bold *p*-values indicate statistical significance. RF, rod fracture; PLIF, posterior lumbar interbody fusion; LLIF, lateral lumbar interbody fusion; ACR, anterior column realignment; PF, posterior fusion; PSO, pedicle subtraction osteotomy.

## Data Availability

The data underlying this article cannot be shared publicly to protect the privacy of the individuals who participated in this study. The data can be shared upon reasonable request to the corresponding authors.

## Abbreviations

RF, rod fracture; ASD, adult spinal deformity; OR, odds ratio; PSO, pedicle subtraction osteotomy; IRB, institutional review board; PI-LL, pelvic incidence minus lumbar lordosis; PT, pelvic tilt; TK, thoracic kyphosis; C7–SVA, C7–sagittal vertical axis; CT, computed tomography; ASA, American Society of Anesthesiologists; BMI, body mass index; PLIF, posterior lumbar interbody fusion; LLIF, lateral lumbar interbody fusion; ACR, anterior column realignment; PF, posterior fusion; SS, sacral slope; T1PA, T1 pelvic angle; C7–CSVL, C7–center sacral vertical line; SRS, Scoliosis Research Society; GAP, Global Alignment and Proportion; SD, standard deviation; UIV, uppermost instrumented vertebra; LIV, lowest instrumented vertebra.
